# The Determinants of Costs and Length of Stay for Hip Fracture Patients

**DOI:** 10.1371/journal.pone.0133545

**Published:** 2015-07-23

**Authors:** Adriana Castelli, Silvio Daidone, Rowena Jacobs, Panagiotis Kasteridis, Andrew David Street

**Affiliations:** 1 Centre for Health Economics, University of York, Heslington, York, YO10 5DD, United Kingdom; 2 Food and Agricultural Organization of the United Nations, Viale Aventino 1, Rome, 00153 Italy; Heinrich-Heine University, Faculty of Medicine, GERMANY

## Abstract

**Background and Purpose:**

An ageing population at greater risk of proximal femoral fracture places an additional clinical and financial burden on hospital and community medical services. We analyse the variation in i) length of stay (LoS) in hospital and ii) costs across the acute care pathway for hip fracture from emergency admission, to hospital stay and follow-up outpatient appointments.

**Patients and Methods:**

We analyse patient-level data from England for 2009/10 for around 60,000 hip fracture cases in 152 hospitals using a random effects generalized linear multi-level model where the dependent variable is given by the patient’s cost or length of stay (LoS). We control for socio-economic characteristics, type of fracture and intervention, co-morbidities, discharge destination of patients, and quality indicators. We also control for provider and social care characteristics.

**Results:**

Older patients and those from more deprived areas have higher costs and LoS, as do those with specific co-morbidities or that develop pressure ulcers, and those transferred between hospitals or readmitted within 28 days. Costs are also higher for those having a computed tomography (CT) scan or cemented arthroscopy. Costs and LoS are lower for those admitted via a 24h emergency department, receiving surgery on the same day of admission, and discharged to their own homes.

**Interpretation:**

Patient and treatment characteristics are more important as determinants of cost and LoS than provider or social care factors. A better understanding of the impact of these characteristics can support providers to develop treatment strategies and pathways to better manage this patient population.

## Introduction

Proximal femoral fracture (PFF) or hip fracture is one of the commonest reasons for admission to an orthopaedic trauma ward. It is usually caused by a fall suffered by older people with osteoporosis or osteopaenia.

In England around 65,000 PFFs occur each year [1] and the medical and social care costs for hip fracture patients amount to about £2 billion annually [2]. Residents of care and nursing homes account for about 30% of all patients admitted to hospital with a PFF. These patients are usually more frail, more dependent in their daily functioning and are more likely to have cognitive impairments than other patients admitted to hospital.

Projections of the prevalence of hip fracture, based on predictions about population growth and changes in age structure, and changes in the incidence of PFF by age group, show that increasingly older people will be affected by PFF. An ageing population at greater risk of PFF will place an additional burden on hospital and community medical services. If the total number of patients suffering PFF reaches 100,000 annually by 2033 [3–7], assuming mean length of stay remains 20 days, these patients will account for an additional 500,000 bed days in 2033 compared to 2008.

Hip fractures have a major impact on health-related quality of life and, for many patients, bring loss of mobility and independence. Patients experiencing PFF are at increased risk of premature death [8–10] and have higher death rates: about 10% die within a month and about one-third die within the year [2, 3, 11]. Most of these deaths are not, however, due to the hip fracture itself, but to the associated conditions and comorbidities that predominantly affect elderly patients.

Clinical evidence-based guidelines have been developed for hip fracture in the UK [2] which propose protocols around a timely and co-ordinated multi-disciplinary collaborative care approach to improve outcomes for patients with PFF. This encourages orthopaedic surgeons, anaesthetists, ortho-geriatricians and their teams to work more closely together. Because the occurrence of fall and fracture often signals underlying ill health, a comprehensive multidisciplinary approach is required from presentation to subsequent follow-up, including the transition from hospital to community care. NICE guidelines for treatment of PFF include a number of standards: prompt admission to orthopaedic care; surgery within 36 hours and within normal working hours; nursing care aimed at minimising pressure ulcer incidence; routine access to ortho-geriatric medical care; assessment and appropriate treatment to promote bone health; and falls assessment.

Despite the financial burden of PFF, research on the factors that affect its costs and LoS is scarce. Two US studies emphasized the role of co-morbidities [11] and medical and nursing interventions [12] on hospitalization costs; a further study found a positive correlation between the American Society of Anesthesiologists (ASA) classification on length of stay (LoS) and costs, but no effect of co-morbidities and body mass index [13]. We build on this work by analysing variation in costs and LoS in England, and by considering the full acute patient care pathway including care provided in the accident and emergency (A&E) department, the hospital stay and during follow-up outpatient (OP) appointments, and by accounting for the supply of social care locally. We identify which factors are the biggest drivers of costs and LoS among socio-demographic characteristics and clinical conditions of the patient and characteristics of the providers of care.

## Methods

This was a retrospective analysis of previously collected, non-identifiable information, and involved no change in the management of patients. Obtaining individual consent was not feasible, so patient records were anonymized and de-identified prior to analysis. The Health and Social Care Information Centre (HSCIC) handles requests for de-identified data and has a legal responsibility to ensure there is an appropriate legal basis to permit the release and subsequent processing of data, that all necessary approvals are in place, and that organisations have appropriate arrangements and safeguards for secure data handling. The HSCIC approved the release of the Hospital Episode Statistics (http://www.hscic.gov.uk/hes) data to the University of York (Data Re-Use Agreements RU536).

### Design

In order to construct the acute pathway for people suffering hip fracture, we used the Hospital Episode Statistics (HES) database containing details of all admissions to National Health Service (NHS) hospitals in England for the financial year 2009/10. HES comprises three main sources of information: 1) the Accident and Emergency (A&E) dataset, which includes individual records for all A&E attendances; 2) the Admitted Patient Care (APC) dataset, which includes inpatients and day-cases; and 3) the Outpatient (OP) dataset, which is made up of individual records for all outpatient attendances.

Costs are not reported in HES, but all English hospitals have to report their activity and costs to the Department of Health (DoH), applying a standard top-down costing methodology to produce “Reference Costs” (RC) for patients allocated to each Healthcare Resource Group (HRG–the English version of diagnosis related groups) in each of their departments [14]. We mapped these costs to each patient according to the hospital and department in which they were treated and the HRG to which they were allocated for each of the above mentioned datasets, building on the process set out in Laudicella et al [15]. We adjusted reported costs by the market forces factor (MFF), this being an index of geographical variation in the prices of land, buildings, and labour [16], designed to account for unavoidable differences in factor prices incurred by different hospitals.

Following National Institute for Health and Care Excellence (NICE) guidelines [2], the population of interest in this study covered patients aged 18 and older presenting with a primary diagnosis of fragility fracture of the hip. This definition includes patients with the following types of fracture: intracapsular (undisplaced and displaced), or femoral neck fractures, and extracapsular (trochanteric and subtrochanteric). Patients were selected based on one of the following ICD-10 (International Classification of Diseases, Tenth Revision) codes: S720 (neck of femur), S721 (perthrocanteric fracture) and S722 (subtrochanteric). We also retrieved information about procedures based on the Office of Population Censuses and Surveys (OPCS) classification, version 4.5, to identify those patients diagnosed with fracture of femur, part unspecified (S729) who were hospitalized with hip fracture. For those patients with unspecified hip fracture, we used OPCS 4.5 W-codes from W370 to W399 for total prosthetic replacement of hip joint to identify our population.

In order to construct the care pathway, we linked A&E activity and patients selected in the APC dataset by means of the unique individual identifier and by identifying only those A&E episodes that occurred within 24 hours before admission to hospital. We linked OP attendances happening after discharge for the following treatment specialties: i) trauma and orthopaedics, ii) rehabilitation, iii) radiology. Our final dataset consisted of 59,067 patients in 152 hospitals. About 83% of the patients were admitted into hospital via A&E and about a quarter had a recorded follow-up OP visit after being discharged.

### Data

The outcomes of interest are the number of days in hospital and cost of treatment for the full patient pathway.

We considered patient characteristics including age, gender, ethnicity and the level of income deprivation in the patient’s area of residence (where a higher value reflects greater deprivation in the local area). We also controlled for discharge destination. We included proxies for the severity of the fracture, dummy variables indicating the type of fracture and we identify comorbidities for each patient, which we coded as binary variables (0/1), as described in Elixhauser et al [17]. We constructed variables which reflect NICE guidelines including surgery on the same date or day after admission (for surgery within 36 hours) and development of pressure ulcers, as well as other quality and treatment indicators such as whether computed tomography (CT) or imaging was performed, and if the patient was re-admitted within 28 days of discharge. We also controlled for patients undergoing any kind of bone or joint surgical procedure as identified by OPCS 4.5 W-codes.

We considered various hospital characteristics including Foundation Trust status [18] as a marker for hospital performance. Foundation status was first introduced in the English NHS in 2004/05 and it grants hospitals with higher performance on a number of indicators greater managerial and financial autonomy from direct central government control. FTs are allowed to retain surpluses, which can re-invest in improved services for patients and service users. We also controlled for number of beds, teaching status, volume of patients treated for hip fracture, an index reporting imaging activity performed in the hospital and three measures of hospital quality: percentage of patients developing pressure ulcers, percentage of patients who received surgery on the same day or the day after admission, and percentage of patients who received cemented arthroplasties. These capture differences in provider treatment practices after controlling for patient level variation in the same indicators. Provider level data come from the the NHS Information Centre (now the Health and Social Care Information Centre) and have been grossed up to hospital level from information contained in the Hospital Episode Statistics (HES) database.

We also accounted for the supply of social care by measuring the amount and type of services and of registered places in care and nursing homes in the local area using data reported by the Care Quality Commission at Local Authority level [19].

### Model Specification and Analysis

Our modelling approach was motivated by the multilevel structure of the data–patients nested in hospitals–and by the features of the distributions of the two dependent variables: i) the total cost of hip fracture from A&E attendance to inpatient hospital stay to follow-up OP visits and ii) the length of inpatient hospital stays.

Costs were right-skewed: average cost of treatment amounts to £8,247, reaches £23,506 at the 99^th^ centile and some people incur costs exceeding £100,000. To reduce skewness and normalise the distribution, costs are typically modelled as a log-linear regression model where costs are logarithmically transformed. When the error term in the log-transformation model is not normal, the nonparametric Duan smearing estimator [20] is usually applied under the assumption of homoscedasticity: the exponentiated linear predictor is multiplied by a smearing factor which is calculated as the average of the exponentiated least squares residuals. Alternatively, Generalised Linear Models (GLM) are estimated and a (GLM) with log link and gamma distributed errors is the most common specification [21–23].

We estimated three multilevel random intercept models to explain the total cost of hip fracture: a linear model with untransformed costs; a model with log-transformed costs; and a GLM model with log-link and gamma distributed errors. We assessed their relative performance based on R-square (of the regression of costs on predicted costs on the raw scale), Root Mean Square Error (RMSE), and Mean Absolute Prediction Error (MAPE). The GLM model performed slightly better in all three measures with explanatory power exceeding 34% and we focus on this model in what follows.

Our multilevel GLM model for costs was therefore described by the log-link function that relates the mean on the raw cost scale with the covariates:

$$\log[C_{ij}|\beta X_{ij}+u_{j}]=\beta X_{ij}+u_{j},\;i=1,\ldots ,N;j=1,\ldots ,M$$ (1)

and the gamma distribution that specifies the variance to be proportional to the square root of the mean. In (1), C_ij_ is the cost of patient i in hospital j, X_ij_ is the column vector of patient and hospital characteristics, **β** is the parameter vector, and u_j_ is hospital-specific random effects with $u_{j}\sim N(0,\theta )$ and *E*(*u*_*j*_ | **x**_*ij*_) = 0. The random effects represent unobserved heterogeneity and induce dependence between patients nested in hospitals.

The distribution of LOS in our sample is positively skewed. The average LoS is 21 days with a median of 15 days. Several approaches have been used in the literature to model LoS including linear regression on log-transformed LoS [24–26] and Generalized Linear Models (GLM) with a log link and Gaussian, Poisson, negative binomial or gamma distributions to characterize the relationship between the variance and conditional mean [27]. A comprehensive review of conventional estimators for LoS and more innovative approaches is provided by Moran and Solomon [28]. In our preliminary single-level analysis, GLM models performed better than a log-transformation model in terms of Root Mean Square Error, so we decided to proceed with a GLM specification. The modified Park test supported the choice of the Poisson distribution, thus our chosen model was a multilevel Poisson model. In single-level settings, a negative binomial model is often preferred as it relaxes the restrictive equidispersion assumption. However, in multilevel models the problem of overdispersion is moderated by introducing the level 2 random effect. Therefore, length of inpatient hospital stay is modelled as:

$$LOS_{ij}∼P[\exp(\beta X_{ij}+\zeta_{j})]$$ (2)

where *ζ*_*j*_ | *X*_*ij*_ ∼ *N*(0,*ψ*) is a hospital-specific random intercept, independent across hospitals.

The two models share the same log-link function and their coefficient estimates can be interpreted as the percentage change in costs/LoS resulting from a partial change in the predictor variable. The exponentiated coefficients or incidence rate ratios (IRRs) also facilitate a semi-elasticity interpretation, with (IRR-1)*100 estimating the result of a discrete (one percentage point) change in the predictor on the percentage change in costs/LoS. In addition, to explore the effects of explanatory variables on *levels–*costs(£) or LoS (days), we also calculated average marginal effects by differentiating the conditional means and averaging over all observations.

Analyses were performed using Stata, version 13 (StataCorp LP, TX).

## Results

Descriptive statistics for patient characteristics, comorbidities, provider and social care variables are presented in Tables 1 and 2, providing means and percentiles of the distribution.

**Table 1:** Patient characteristics, n = 59067.

Variable	mean	P1	P50	P99
*Dependent variables*				
Full pathway costs (£)	8,247	1,107	7,375	23,506
Inpatient length of stay	20.90	1	15	102
*Demographic*				
Age in years	81.24	41	84	99
Gender: female	0.72	0	1	1
Index of multiple deprivation: income domain	0.15	0.02	0.12	0.51
Other or unknown race [reference]	0.09	0	0	1
White	0.91	0	1	1
*A&E department type attendance*				
Did not attend A&E [reference]	0.17	0	0	1
24 hour/ full resuscitation facilities	0.55	0	1	1
Consultant-led mono specialty	0.01	0	0	0
Other type/minor injury activity	0.01	0	0	1
Department not known	0.26	0	0	1
*Type of fracture*				
Fracture of neck of femur [reference]	0.72	0	1	1
Pertrochanteric fracture	0.24	0	0	1
Subtrochanteric fracture	0.03	0	0	1
Fracture of femur, part unspecified	0.01	0	0	0
*Cause and site of hip fracture*				
Multiple hip fracture	0.01	0	0	0
Injury due to fall	0.76	0	1	1
*Severity of fracture*				
Secondary non-hip injuries	0.004	0	0	0
Any bone or joint surgical procedure	0.92	0	1	1
*Quality and treatment indicators*				
Surgery same date or day after admission	0.74	0	1	1
Arthroplasties cemented	0.27	0	0	1
Pressure ulcers	0.02	0	0	1
Use of epidural anaesthetic	0.005	0	0	0
Patient had computed tomography	0.05	0	0	1
Patient had other type of imaging	0.01	0	0	0
Patient readmitted within 28 days	0.08	0	0	1
Patient readmitted after 28 days	0.02	0	0	1
Patient transferred between providers	0.21	0	0	1
Outpatient attendances after discharge	0.46	0	0	5
*Discharge destination*				
Usual residence [reference]	0.54	0	1	1
Temporary residence	0.03	0	0	1
Other provider	0.14	0	0	1
Nursing home, residential and LA care	0.09	0	0	1
Patient died	0.07	0	0	1
Other destination	0.13	0	0	1

**Notes**: Also included but omitted for brevity are dummy variables for co-morbidities. *Other destination* includes psychiatric/penal hospital, non-NHS run hospital-medium secure unit, and unknown destination. P1, P50, P99 are 1%, 50%, 99% percentiles respectively.

**Table 2:** Provider level and social care variables.

	mean	P1	P50	P99
*Provider level*, *n = 152*				
Number of beds	768	65	695	2,073
Teaching hospital	0.17	0	0	1
Foundation Trust status	0.50	0	0.5	1
Number of hip fracture patients	392	1	376	916
% with cemented arthroplasties	27	0	26	50
% developing pressure ulcers	3	0	2	15
% early surgery	73	18	75	100
Imaging index	8	0	7	22
Social care variables, n = 95				
Total number of agency services in 1,000	7	2	7	13
Total number of home places in 10,000	49	12	45	107

**Note**: P1, P50, P99 are 1%, 50%, 99% percentiles respectively.

Marginal effects for both cost and LoS analyses are reported in Table 3. IRRs are provided in S1 Table. The variances of the hospital specific random effects for the costs and LoS models are respectively 28.53 (95% CI = [23.60, 33.93]) and 0.057 (95% CI = [0.045, 0.072]).

**Table 3:** Marginal effects for costs (full pathway) and length of stay (inpatient), n = 59,067.

	Costs (in GBP)		LOS	
	Marg Eff	95% CI	Marg Eff	95% CI
**Patient characteristics**				
*Demographic*				
Age in years	34***	(30, 38)	0.28***	(0.27, 0.29)
Female	208	(-155, 571)	-0.71***	(-0.80, -0.62)
IMD (income domain)	642***	(400, 884)	4.31***	(3.91, 4.71)
White	33	(-56, 122)	0.71***	(0.57, 0.85)
Other or unknown race [reference]				
*A&E attendance*				
Did not attend A&E [reference]				
24 hour/ full resuscitation facilities	-367***	(-459, -275)	-7.14***	(-7.47, -6.81)
Consultant-led mono specialty	433	(-1, 866)	-11.39***	(-12.28, -10.50)
Other type/minor injury activity	-524*	(-943, -105)	-8.39***	(-9.18, -7.60)
Department not known	-203**	(-330, -76)	-7.81***	(-8.19, -7.43)
*Type of fracture*				
Fracture of the neck of femur [reference]				
Pertrochanteric fracture	159***	(95, 222)	0.49***	(0.39, 0.58)
Subtrochanteric fracture	570***	(431, 708)	2.67***	(2.44, 2.90)
Fracture of femur, part unspecified	721***	(445, 997)	3.38***	(2.97, 3.79)
*Site and cause of hip fracture*				
Fracture of the neck of femur [reference]				
Multiple hip fracture	2582***	(2232, 2931)	5.39***	(4.94, 5.85)
Injury due to fall	-2663***	(-2752, -2574)	-5.31***	(-5.56, -5.06)
*Severity of fracture*				
Fracture of the neck of femur [reference]				
Secondary non-hip injuries	3650***	(3254, 4046)	10.33***	(9.76, 10.91)
Any bone or joint surgical procedure	5864***	(5716, 6012)	8.79***	(8.38, 9.20)
*Quality & treatment indicators*				
Surgery same date or day after admission	-655***	(-723, -587)	-5.87***	(-6.13, -5.60)
Arthroplasties cemented	418***	(355, 481)	-0.06	(-0.15, 0.03)
Pressure ulcers	1943***	(1779, 2106)	8.42***	(8.02, 8.82)
Use of epidural anaesthetic	-189	(-566, 188)	-0.11	(-0.66, 0.43)
Patient had computed tomography	1109***	(993, 1226)	5.13***	(4.86, 5.39)
Patient had other type of imaging	415**	(131, 700)	1.60***	(1.21, 2.00)
Patient readmitted within 28 days	228***	(136, 319)	0.99***	(0.85, 1.13)
Patient readmitted after 28 days	-271*	(-434, 107)	-1.65***	(-1.91, -1.39)
Patient transferred between providers	382***	(259, 504)	1.94***	(1.75, 2.13)
Outpatient attendances after discharge	35**	(10, 59)	-0.82***	(-0.87, -0.76)
*Co-morbidities*				
None [reference]				
Congestive heart failure	346***	(247, 446)	1.99***	(1.83, 2.15)
Cardiac arrhythmias	168**	(50, 285)	1.04***	(0.87, 1.21)
Valvular diseases	183**	(52, 314)	0.64***	(0.45, 0.82)
Pulmonary circulation disorders	545***	(316, 773)	2.44***	(2.12, 2.75)
Peripheral vascular disorders	286***	(130, 442)	1.74***	(1.52, 1.96)
Hypertension	82**	(31, 133)	0.30***	(0.23, 0.38)
Paralysis	713***	(512, 914)	4.32***	(4.00, 4.65)
Neurological disorders	659***	(563, 754)	5.17***	(4.91, 5.43)
Chronic pulmonary disease	285***	(215, 355)	0.64***	(0.54, 0.75)
Diabetes, uncomplicated	268***	(191, 344)	1.64***	(1.51, 1.77)
Diabetes, complicated	1385***	(1072, 1698)	4.75***	(4.29, 5.20)
Hypothyroidism	75	(-16, 167)	0.24***	(0.10, 0.37)
Renal failure	714***	(612, 816)	1.92***	(1.76, 2.07)
Liver disease	347*	(81, 612)	3.41***	(3.02, 3.79)
Peptic ulcer disease excluding bleeding	1361***	(934, 1788)	7.04***	(6.47, 7.60)
Lymphoma	415*	(39, 791)	2.47***	(1.94, 3.00)
Metastatic cancer	-10	(-247, 227)	-0.51**	(-0.85, -0.17)
Solid tumor without metastasis	206**	(59, 353)	0.66***	(0.45, 0.86)
Rheumatoid arthritis	218**	(84, 353)	0.66***	(0.46, 0.87)
Coagulopathy	747***	(365, 1129)	2.87***	(2.35, 3.40)
Obesity	595**	(241, 950)	3.08***	(2.58, 3.58)
Weight loss	454*	(102, 805)	4.06***	(3.62, 4.50)
Fluid and electrolyte disorders	978***	(872, 1083)	4.76***	(4.52, 5.00)
Blood loss anaemia	1138**	(420,1856)	3.17***	(2.28, 4.06)
Deficiency anaemia	392***	(235, 550)	2.29***	(2.06, 2.52)
Alcohol and drug abuse	345***	(189, 502)	3.00***	(2.73, 3.27)
Psychoses	777***	(479, 1076)	4.94***	(4.49, 5.38)
Depression	381***	(242, 520)	3.39***	(3.16, 3.63)
*Discharge destination*				
Usual residence [reference]				
Temporary residence	442***	(300, 584)	3.44***	(3.20, 3.68)
Other provider	-398***	(-533, -264)	-3.39***	(-3.64, -3.15)
Nursing home, residential and LA care	1125***	(1031, 1220)	7.93***	(7.57, 8.29)
Patient died	65	(-40, 169)	-4.59***	(-4.84, -4.33)
Other destination	-440***	(-523, -356)	5.21***	(4.96, 5.46)
**Provider characteristics**				
# beds	1***	(1, 2)	0.01***	(0.00, 0.01)
Teaching hospital	-1033***	(-1433, -633)	-2.31	(-5.15, 0.53)
Foundation Trust status	-613**	(-1005, -222)	0.60	(-1.00, 2.21)
# hip fracture patients	-6***	(-7, -5)	-0.01**	(-0.02, -0.002)
% with cemented arthroplasties	-8	(-20, 4)	0.003	(-0.06, 0.07)
% developing pressure ulcers	-108***	(-148, 68)	0.06	(-0.24, 0.35)
% early surgery	28***	(15, 42)	0.05	(-0.02, 0.12)
Imaging index	-18	(-49, 14)	-0.14	(-0.34, 0.06)
**Social care variables**				
Total # agency services in 1,000	-6	(-31, 19)	0.17***	(0.13, 0.20)
Total # home places in 10,000	0	(-4, 3)	-0.03***	(-0.03, -0.02)

* p< 0.05

** p < 0.01

*** p < 0.001

### Patient characteristics

#### Demographics

Of the 59,067 patients in our sample, most patients were female (72%) and white (91%). The average age was 81 years and was positively related to higher total costs of hip fracture care and LoS. Patients residing in areas with higher income deprivation had higher total costs and stayed longer in hospital. Men and white ethnicity had longer LoS, but these characteristics did not appear to be cost drivers.

#### A&E attendance

83% of patients were admitted into hospital through an A&E department. Compared to patients admitted directly to hospital, and not entering via an A&E department, patients stayed on average fewer days in hospital. For instance, those admitted through a consultant led A&E department had on average about 11 days shorter stays. However, patients admitted through a consultant led A&E department did not have significantly lower costs.

#### Type of fracture

Fracture of the neck of femur was the commonest type of injury (72%), followed by pertrochanteric facture (24%). Compared to fracture of the neck of femur, pertrochanteric, subtronchanteric, and unspecified fracture of femur were more costly and had higher LoS.

#### Cause and site of hip fracture

About 76% of hip fractures happened after a fall and these patients tended to be less costly (by £2,663) and have shorter LoS (by 5 days) than those who did not suffer a fall. Treatment for patients with multiple hip fractures cost £2,582 more than fracture of the neck of femur and required 5 days longer in hospital.

#### Severity of fracture

Patients who had any bone or joint surgical procedure (92%) were more costly by £5,864 and stayed about 9 days longer in hospital compared to those who did not undergo any procedure. A limited number of patients were diagnosed with secondary injuries not related to the hip. Treatment for these patients added £3,650 to costs and 10 days to LoS.

#### Quality & treatment indicators

Patients that received surgery on the same date or the day after admission (74%) had lower costs and LoS. Costs were higher for patients undergoing cemented arthroplasty, but there was no effect on LoS. Pressure ulcers and CT scans were associated with increased costs of £1,943 and £1,109 and LoS by 8 and 5 days respectively. 8% of patients experienced an emergency readmission within 28 days and 21% were transferred between providers, both of which drive costs and LoS up. The initial admission for patients who were subsequently readmitted may be more costly and have longer LoS because of their casemix complexity. On average, patients have 0.46 subsequent outpatient attendances but these did not increase costs significantly, and actually were associated with reduced LoS by almost 1 day.

#### Comorbidities

**Fig 1:**
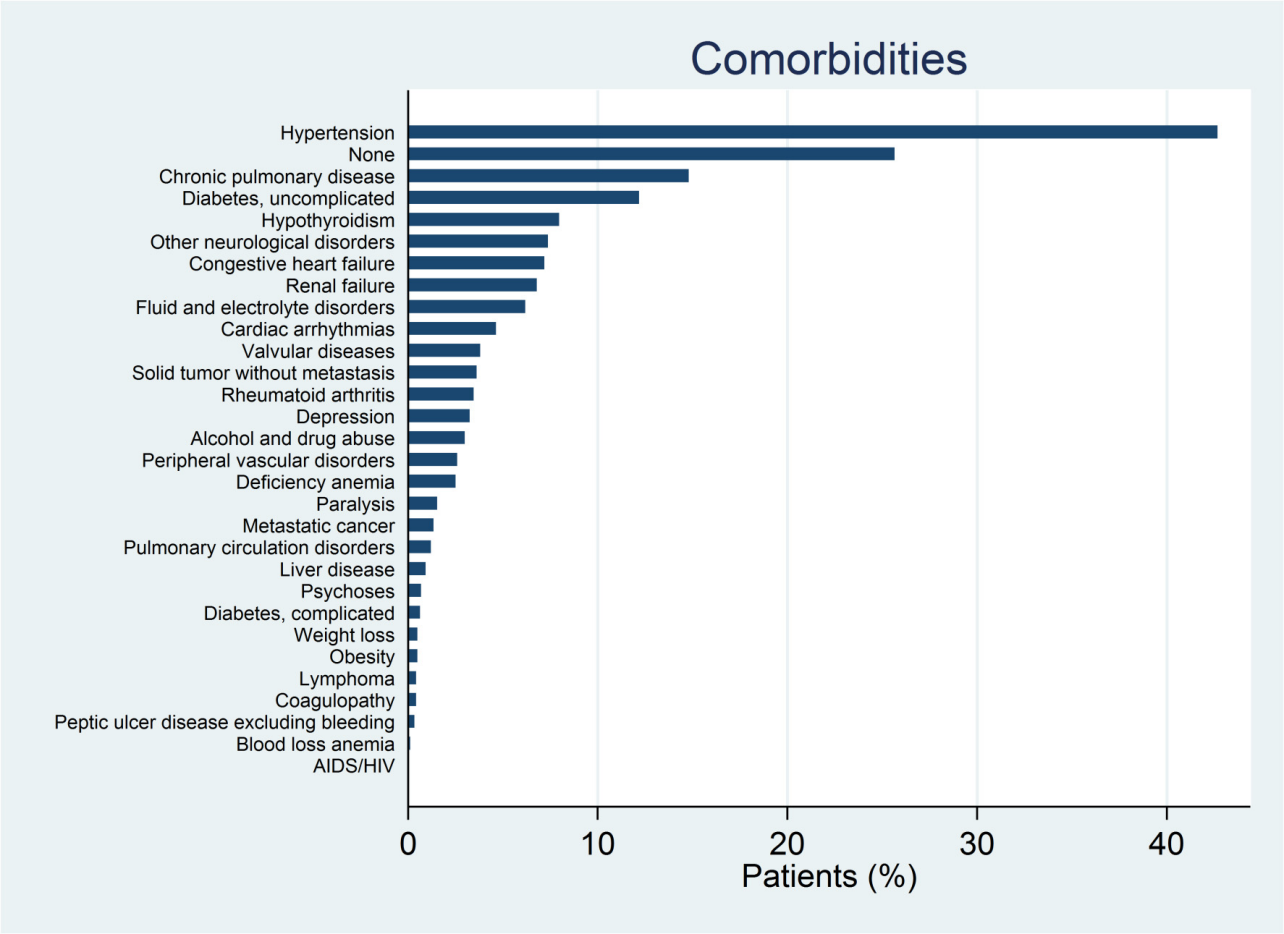
Prevalence of comorbidities in the cohort of patients.

Fig 1 shows the prevalence of comorbidities in our cohort of patients. Hypertension was the main comorbidity, affecting more than 40% of patients, followed by chronic pulmonary disease (14%) and diabetes (12%). Almost a quarter of the sample did not appear to have other coexisting medical conditions not directly related to the principal diagnosis.

Of the 28 comorbidities, 16 had a statistically significant impact on costs. Despite being the most common co-morbidity, hypertension added very little to costs.

Diabetes with chronic complications (£1,385), peptic ulcer disease (£1,361), fluid and electrolyte disorders (£978), psychoses (£777), coagulopathy (£747), renal failure (£714), and paralysis (£713) were the most costly comorbidities.

With the exception of metastatic cancer, all comorbidities increased LoS. The biggest drivers of LoS were peptic ulcers, neurological disorders, psychoses, fluid and electrolyte disorders, diabetes with complications, paralysis and weight loss, all addingbetween 4 and 7 days to LOS.

#### Discharge destination

More than half of patients were discharged to their usual residence, whilst almost 14% were discharged to another provider and 7% died in hospital. Patients discharged to a nursing home stayed 8 days longer in hospital than those discharged to their usual residence. In-hospital death and being discharged to other providers was associated with 5 and 3 days shorter LoS respectively.

### Provider characteristics

Care for hip fracture was generally provided in fairly large hospitals (average 768 beds). About half of the hospitals had Foundation Trust status (implying better performance) and less than a fifth were teaching hospitals. On average, almost 400 hip fracture patients were treated in each hospital per year, even though there was a lot of variability (from 1 to 1,014 patients). 73% of patients had surgery the same day or the day after admission and more than a quarter received cemented arthroplasties. Less than 3% developed pressure ulcers and imaging was recorded for only 7% of patients. The imaging index is a measure of complexity which is driven by the cost of each procedure.

Serving a larger number of PFF patients had a very small impact on costs, as did size (number of beds), early surgery and developing pressure ulcers. Teaching status was associated with reduced costs (£1,033). None of the provider characteristics affected LoS.

### Social care

The supply of social care in the local area appeared not to have a significant influence on health care costs and the impact on LoS was marginal.

## Discussion

This paper has examined variation in acute care costs and LoS for almost 60,000 people treated in 152 English hospitals after suffering proximal femoral fracture (PFF). Consistent with Nikkel et al [11], we found that co-morbidities such as diabetes, peptic ulcer disease, fluid and electrolyte disorders, psychoses, coagulopathy, renal failure and paralysis were important determinants of costs and LoS for hip fracture, increasing LoS by up to 7 days. Other studies found a different set of co-morbidities to be determinants of cost. Nikkel et al [11] found weight loss, pulmonary circulation disorders, and congestive heart failure to be major cost drivers but in this study these had only moderate or insignificant effects. Despite being the most common co-morbidity, hypertension added very little to costs while Nikkel et al [11] found that hypertension was associated with slightly lower costs. We found that men and white ethnicity had longer LoS, but these characteristics did not appear to be cost drivers as found by others [12].

Similarly to Nikkel et al [11], we found that receiving surgery within the first 48 hours of admission significantly reduced costs (by £655) and LoS of PFF patients by almost 6 days. This underscores the benefits which may accrue from implementation of NICE guidelines around surgery “on the day of, or the day after, admission” [2].

We also found that LoS was 11 days shorter for those admitted through a consultant led A&E department. It is recognised that immediate diagnosis is crucial in avoiding delayed surgery and later complications and admission through an A&E department may be more effective in realising timely diagnosis and treatment of patients [2].

We also found that subsequent outpatient attendances did not increase costs significantly, but reduce LoS by almost 1 day. This may be because early discharge of patients is linked to a structured plan of rehabilitation which may include outpatient attendances.

A limitation was that our pathway remains incomplete. We were not able to track patients that received treatment for PFF from other healthcare providers subsequent to their discharge and we were unable to include social care costs directly in any of our models as these were not available at patient level. Instead we included measures of the local supply of care homes and nursing homes in our models to capture the impact of differences in social care provision across the country. We anticipated that care received in other hospital providers or in care homes and nursing homes will add substantially to the total costs of treating patients with hip fracture; therefore, our analysis likely underestimated the true costs to the NHS in England of patients with PFF. A further drawback is that costs are based on Reference Cost data that do not capture precisely the costs of care for each individual patient. Rather all English hospitals are required to apply a standard top-down costing methodology, with costs allocated to patients in a similar way to the charge data reported to the Healthcare Cost Report Information System (HCRIS) (see http://www.cms.gov/Medicare-Fee-for-Service-Payment/AcuteInpatientPPS/FY2015-IPPS-Final-Rule-Home-Page-Items/FY2015-Final-Rule-Data-Files.html) and used in many studies of hospital costs (e.g. see [29] for a review). The allocation rules mean that the Reference Cost data exhibit less variation than occurs in reality. Recognising this limitation we also analysed variation in LoS, which is measured accurately for each patient. Another limitation is that surgeon volume is likely to be an important confounder. However, to construct this variable we would need to use the consultant code variable in the HES database, which is a sensitive field, to which we did not have access.

Despite these limitations, this work added to current knowledge by articulating the most important drivers of cost and LoS in the acute care pathway for patients with PFF. Previous analyses [11, 12] of pathway costs did not include emergency (A&E) attendances, outpatient visits, or availability of social care. Through linking of three large datasets we were able to add these components. We also considered the role of a number of best practice standards captured in the NICE guidance on the treatment of PFF such as prompt surgery and minimising pressure ulcer incidence. The patient and treatment characteristics were far more important as determinants of cost and LoS than provider or social care factors. A better understanding of the impact of these characteristics can support providers to develop treatment strategies and pathways to better manage this patient population.

## Supporting Information

S1 Table Incidence Rate Ratios (IRR) for costs (full pathway) and length of stay (inpatient), n = 59,067. (DOCX)
