# Supplementary material for: The Determinants of Costs and Length of Stay for Hip Fracture Patients
Source: PLoS One. 2015 Jul 23;10(7):e0133545. doi: 10.1371/journal.pone.0133545 (PMC4512684; doi:10.1371/journal.pone.0133545)
Supplement: S1 Table — (DOCX) [file pone.0133545.s001.docx]

**S1 Table: Incidence Rate Ratios (IRR) for costs (full pathway) and length of stay (inpatient), n=59,067**

|  | Costs (in GBP) | | LOS | |
| --- | --- | --- | --- | --- |
|  | IRR | 95% CI | IRR | 95% CI |
| **Patient characteristics** |  |  |  |  |
| *Demographic* |  |  |  |  |
| Age in years | 1.004*** | (1.004, 1.005) | 1.015*** | (1.014, 1.015) |
| Female | 1.026 | (0.981, 1.072) | 1.065*** | (1.033, 1.098) |
| IMD (income domain) | 1.081*** | (1.05, 1.114) | 1.233*** | (1.212, 1.254) |
| Other or unknown race [reference] | 1.004 | (0.993, 1.015) | 1.035*** | (1.028, 1.042) |
| White | 1.004*** | (1.004, 1.005) | 1.015*** | (1.014, 1.015) |
| *A&E attendance* | | | | |
| Did not attend A&E [reference] |  |  |  |  |
| 24 hour/ full resuscitation facilities | 0.956*** | (0.946, 0.967) | 0.707*** | (0.703, 0.711) |
| Consultant-led mono specialty | 1.054 | (0.999, 1.112) | 0.575*** | (0.555, 0.596) |
| Other type/minor injury activity | 0.938* | (0.891, 0.987) | 0.665*** | (0.643, 0.688) |
| Department not known | 0.976** | (0.961, 0.991) | 0.684*** | (0.678, 0.690) |
| *Type of fracture* | | | | |
| Fracture of the neck of femur [reference] |  |  |  |  |
| Pertrochanteric fracture | 1.019*** | (1.012, 1.027) | 1.024*** | (1.019, 1.029) |
| Subtrochanteric fracture | 1.072*** | (1.054, 1.09) | 1.138*** | (1.128, 1.150) |
| Fracture of femur, part unspecified | 1.092*** | (1.056, 1.129) | 1.178*** | (1.157, 1.201) |
| *Site and cause of hip fracture* |  |  |  |  |
| Multiple hip fracture | 1.370*** | (1.314, 1.43) | 1.300*** | (1.275, 1.325) |
| Injury due to fall | 0.722*** | (0.716, 0.729) | 0.772*** | (0.769, 0.776) |
| *Severity of fracture* | | | | |
| Secondary non-hip injuries | 1.561*** | (1.489, 1.637) | 1.652*** | (1.623, 1.682) |
| Any bone or joint surgical procedure | 2.046*** | (2.019, 2.073) | 1.533*** | (1.522, 1.545) |
| *Quality & treatment indicators* | | | | |
| Surgery same date or day after admission | 0.923*** | (0.916, 0.931) | 0.752*** | (0.749, 0.755) |
| Arthroplasties cemented | 1.052*** | (1.044, 1.06) | 0.997 | (0.993, 1.002) |
| Pressure ulcers | 1.268*** | (1.243, 1.293) | 1.506*** | (1.493, 1.519) |
| Use of epidural anaesthetic | 0.977 | (0.933, 1.023) | 0.994 | (0.968, 1.021) |
| Patient had computed tomography | 1.145*** | (1.129, 1.161) | 1.283*** | (1.274, 1.292) |
| Patient had other type of imaging | 1.052** | (1.016, 1.089) | 1.081*** | (1.061, 1.102) |
| Patient readmitted within 28 days | 1.028*** | (1.017, 1.04) | 1.049*** | (1.043, 1.056) |
| Patient readmitted after 28 days | 0.968* | (0.948, 0.987) | 0.923*** | (0.912, 0.934) |
| Patient transferred between providers | 1.048*** | (1.032, 1.063) | 1.099*** | (1.090, 1.108) |
| Outpatient attendances after discharge | 1.004** | (1.001, 1.007) | 0.961*** | (0.959, 0.963) |
| *Co-morbidities* | | | | |
| None [reference] |  |  |  |  |
| Congestive heart failure | 1.043*** | (1.031, 1.056) | 1.101*** | (1.094, 1.109) |
| Cardiac arrhythmias | 1.021** | (1.006, 1.035) | 1.052*** | (1.043, 1.060) |
| Valvular diseases | 1.023** | (1.006, 1.039) | 1.031*** | (1.022, 1.040) |
| Pulmonary circulation disorders | 1.069*** | (1.039, 1.099) | 1.126*** | (1.110, 1.142) |
| Peripheral vascular disorders | 1.036*** | (1.016, 1.055) | 1.088*** | (1.077, 1.099) |
| Hypertension | 1.010** | (1.004, 1.016) | 1.015*** | (1.011, 1.019) |
| Paralysis | 1.091*** | (1.064, 1.118) | 1.234*** | (1.218, 1.250) |
| Neurological disorders | 1.084*** | (1.071, 1.096) | 1.286*** | (1.278, 1.294) |
| Chronic pulmonary disease | 1.035*** | (1.026, 1.044) | 1.032*** | (1.027, 1.037) |
| Diabetes, uncomplicated | 1.033*** | (1.024, 1.043) | 1.083*** | (1.077, 1.089) |
| Diabetes, complicated | 1.184*** | (1.14, 1.23) | 1.259*** | (1.235, 1.284) |
| Hypothyroidism | 1.009 | (0.998, 1.021) | 1.012*** | (1.005, 1.018) |
| Renal failure | 1.091*** | (1.078, 1.105) | 1.098*** | (1.090, 1.105) |
| Liver disease | 1.043* | (1.01, 1.077) | 1.180*** | (1.160, 1.200) |
| Peptic ulcer disease excluding bleeding | 1.181*** | (1.121, 1.244) | 1.408*** | (1.376, 1.441) |
| Lymphoma | 1.052* | (1.005, 1.101) | 1.128*** | (1.099, 1.156) |
| Metastatic cancer | 0.999 | (0.97, 1.028) | 0.975** | (0.959, 0.992) |
| Solid tumor without metastasis | 1.026** | (1.007, 1.044) | 1.033*** | (1.022, 1.043) |
| Rheumatoid arthritis | 1.027** | (1.01, 1.044) | 1.033*** | (1.023, 1.043) |
| Coagulopathy | 1.095*** | (1.045, 1.148) | 1.150*** | (1.122, 1.179) |
| Obesity | 1.075** | (1.03, 1.123) | 1.161*** | (1.135, 1.189) |
| Weight loss | 1.057* | (1.013, 1.103) | 1.218*** | (1.194, 1.242) |
| Fluid and electrolyte disorders | 1.127*** | (1.112, 1.141) | 1.260*** | (1.252, 1.268) |
| Blood loss anaemia | 1.149** | (1.053, 1.254) | 1.167*** | (1.118, 1.218) |
| Deficiency anaemia | 1.049*** | (1.029, 1.069) | 1.118*** | (1.107, 1.129) |
| Alcohol and drug abuse | 1.043*** | (1.023, 1.063) | 1.157*** | (1.144, 1.170) |
| Psychoses | 1.100*** | (1.06, 1.14) | 1.271*** | (1.247, 1.296) |
| Depression | 1.048*** | (1.03, 1.066) | 1.179*** | (1.169, 1.190) |
| *Discharge destination* | | | | |
| Usual residence [reference] |  |  |  |  |
| Temporary residence | 1.055*** | (1.037, 1.074) | 1.182*** | (1.171, 1.193) |
| Other provider | 0.953*** | (0.937, 0.968) | 0.848*** | (0.840, 0.856) |
| Nursing home, residential and LA care | 1.147*** | (1.134, 1.16) | 1.470*** | (1.462, 1.478) |
| Patient died | 1.008 | (0.995, 1.021) | 0.800*** | (0.794, 0.806) |
| Other destination | 0.948*** | (0.938, 0.957) | 1.288*** | (1.281, 1.295) |
| **Provider characteristics** | | | | |
| # beds | 1.000*** | (1, 1) | 1.000*** | (1.000, 1.001) |
| Teaching hospital | 0.882*** | (0.84, 0.925) | 0.894 | (0.779, 1.026) |
| Foundation Trust status | 0.928** | (0.885, 0.973) | 1.030 | (0.952, 1.113) |
| # hip fracture patients | 0.999*** | (0.999, 0.999) | 1.000** | (0.999, 1.000) |
| % with cemented arthroplasties | 0.908 | (0.787, 1.047) | 1.000 | (0.997, 1.003) |
| % developing pressure ulcers | 0.268*** | (0.167, 0.431) | 1.003 | (0.988, 1.017) |
| % early surgery | 1.411*** | (1.196, 1.665) | 1.003 | (0.999, 1.006) |
| Imaging index | 0.998 | (0.994, 1.002) | 0.993 | (0.984, 1.003) |
| Social care variables | | | | |
| Total # agency services in 1,000 | 0.999 | (0.996, 1.002) | 1.008*** | (1.006, 1.010) |
| Total # home places in 10,000 | 1.000 | (1.000, 1.000) | 0.999*** | (0.999, 0.999) |

* p< 0.05, ** p < 0.01, *** p < 0.001
